# Perspective of Using Magnesium Oxychloride Cement (MOC) and Wood as a Composite Building Material: A Bibliometric Literature Review

**DOI:** 10.3390/ma15051772

**Published:** 2022-02-26

**Authors:** Andreea Maier, Daniela Lucia Manea

**Affiliations:** Faculty of Civil Engineering, Technical University of Cluj-Napoca, 400114 Cluj-Napoca, Romania; daniela.manea@ccm.utcluj.ro

**Keywords:** wood, magnesium oxychloride (MOC) cement, compatibility wood–cement, wood–cement building materials

## Abstract

The building industry is known as one of the biggest consumers of natural resources and an important producer of CO_2_ emissions. The biggest greenhouse gas emissions are recorded in the production of cement and metallic building materials. The purpose of this paper is to investigate if magnesium oxychloride cement (MOC) can be used as an alternative to the ordinary Portland cement in the mixture of wood–cement composite building materials in order to decrease the negative impact of the construction industry on the environment. The research methodology includes bibliometric literature research, a scientometric analysis and an in-depth discussion. The data used for the research were obtained by interrogating the ISI Web of Science database, selected using the guidelines of the PRISMA method and processed with the help of VOSviewer and Bibliometrix software. The research results indicate an increasing interest in this topic; for example, in the last five years, three times more articles were published on the subject of MOC cement than the number of all articles collected in previous years. Compared to ordinary Portland cement, MOC cement presents a good match with wood, so MOC can be a substitute for ordinary cement to manufacture wood-cement particleboard, especially for the wood species that have high incompatibility with ordinary cement.

## 1. Introduction

The current rhythm of social development is starting to have visible effects on the environment. In the last years, all over the world, leaders have been trying to adopt and implement solutions to decrease the impact of human activities on the environment. The UN Climate Change Conference COP26 in Glasgow, in November 2021, adopted a ten-year work program [1] to intensify the efforts of implementing the goals of Actions for Climate Empowerment (ACE) such that all members of the society to engage in climate actions [2]. One of the biggest challenges in this effort is related to decreasing greenhouse emissions and stopping the effects of climate change. 

The construction industry is one of the biggest producers of CO_2_ emissions, mainly because of the embodied and operational energy use. The biggest greenhouse gas emissions are recorded in the production of cement and metallic building materials. Considering that all over the world, billions of metric tons of Portland cement are produced, it can be seen the environmental burden and the huge amount of CO_2_ gases released in the atmosphere in the process of limestone decomposition and coal burning [3]. 

The pressure on the construction industry to produce more living and working spaces are felt especially in the case of big cities where, given the awareness of higher living standard, more and more peoples are choosing to move there and thus cities are developing and growing their size. In a report of the United Nations [4], it was shown that from the total world population, half of them live in cities, and some of the projections indicate an increase of 60% for the urban population until 2030. By taking into account that the living and working requirements are constantly changing, it becomes obvious that the solution to decrease the negative impact of constructions on the environment by decreasing the production of the building is not feasible. 

This awareness of the environmental issues generated by the construction industry has boosted the search for new and greener solutions to construction; thus, the importance of renewable building materials increased substantially in the last years. Wood as a building material is one of this type, and the interest in knowing and developing new ways to use it in the construction process is becoming more and more important. As shown in [4], wood as a building material has many ecological advantages, such as its ability to store carbon, and economic benefits, such as lower energy costs for processing the possibility of prefabrication and multiple possibilities of using waste generated in all processes.

As each building material has advantages and disadvantages, the idea of totally replacing the concrete buildings with wood buildings is not possible to be implemented. A better approach is to combine the advantages of concrete and wood to create a more sustainable building material. This can be performed by crushing the wood in small parts so it can be introduced into the concrete mixture to decrease the amount of cement. If in this equation enters another big challenge of the construction industry, the waste management issues [5], and thus to further use the wood waste in the concrete composition, the premises for finding a good solution to the environmental problems appear.

The greatest difficulty in realizing wood–cement building materials is the incompatibility between wood and cement [6]. This is caused by the blocking or stopping the hydration process of cement by some of the soluble chemicals in the wood [7,8]. The result is obtaining wood–cement composites with lower mechanical strength compared with the simple cement products. A solution to overcome this incompatibility is the use to use other alternatives in the wood–cement composites such as magnesia-based cements [9]. As it has lower alkalinity and a short setting time, MOC cement can be a perfect match for wood–cement products. At the same time, the natural color of the MOC cement is yellowish, so it is closer to the color of many natural wood species. 

Magnesium oxychloride cement (MOC) is one of the alternatives, also known as Sorel cement, this type of cement is formed by the reaction of light burned MgO with MgCl_2_ water solution [8]. The reduced environmental impact is given by the lower calcination temperature, somewhere around 700 to 1000 °C, compared to 1400 to 1450 °C in the case of Portland cement. MOC cement’s biggest potential can be its capability to be used as a component of low-energy building composite materials while acting as a CO_2_ sink [10]. 

### Research Purpose

Given the above-described context, the purpose of this research was to investigate if the use of MOC cement is a good solution to replace the Portland cement in the mixture of wood and cement composite building materials, thus eliminating all the incompatibility issues between wood and cement. The main research questions that this study is trying to answer can be formulated as follows: “Is the MOC cement a good solution to be used in wood cement composite building materials in order to reduce the negative impact of the construction industry on the environment?”

The search began with the identification of the main concerns related to this topic in the literature. The data used in the analysis were obtained from the ISI Web of Science database. The method used to extract and select the best data was the PRISMA (Preferred Reporting Items for Systematic Reviews and Meta Analyses) method. 

The structure of the paper follows the structure of a scientific review paper. It starts with a short introduction and a short background presentation of the MOC cement. The presentation of the main results obtained from conducting the research together with a short discussion part after each result is the next part of the paper. The responses to the research question are presented in the discussions part of the article. The paper ends with the presentation of the main conclusions and with the list of bibliographic titles used in this study.

## 2. Background of Magnesium Oxychloride Cement (MOC)

The discovery of the Magnesium oxychloride cement (MOC) was made more than 150 years ago, in 1867, by a French civil engineer, inventor and chemist named Stanislas Sorel. From the name of the inventor, the MOC cement is also called Sorel cement [11]. MOC belongs to a special type of cement, characterized by rapid setting, strong binding ability and high strength in normal air condition [12]. The main components of MOC are caustic burned magnesia powder and magnesium chloride hexahydrate (MgCl_2__6H_2_O) mixed under ambient temperature and pressure [13]. The main stable oxychlorides at ambient temperature are the so-called “phase 3” and “phase 5”, whose formulas can be written as 3Mg(OH)_2_·MgCl_2_·8H_2_O and 5Mg(OH)_2_·MgCl_2_·8H_2_O, respectively, or, equivalently, Mg_2_(OH)_3_Cl_4_H_2_O and Mg_3_(OH)_5_Cl 4H_2_O [14].

MOC cement attracted much attention due to the fact that, in many ways, it exhibits properties superior to standard Portland cement, and among the low-carbon cement materials, it exhibits considerable potential in replacing Portland cement [15]. The main advantages that make the MOC cement superior to ordinary cement are referring to lower CO_2_ emission, short setting time, the lack of humid curing, low alkalinity and excellent mechanical strength [16]. Because the phase composition could be regulated through adjusting the molar ratio of precursor [17,18], MOC cement provides a series of promising cementitious substrates [19]. Furthermore, it exhibits rheological properties that enable the material to flow into irregular cavities [20]. It also exhibits comparatively high flexural strength (≥4 MPa), compressive strength (≥69 MPa) and elastic modulus (70–85 GPa) [21].

Due to the superior properties, such as high strength at the early stage, outstanding thermal insulation [22] and fire resistance [23], and distinguished resistance to abrasion, MOC has been successfully used as environmental friendly fireproof thermal insulation products, urban refuse/cement compound floor tiles and lightweight boards [24].

The major commercial applications are in industrial flooring, fire protection, grinding wheels and wall panels [25]. MOC boards have some superior properties compared with gypsum or fiber-based boards, such as greater fire resistance, lower thermal conductivity, improved resistance to abrasion and greater strength. The versatility of magnesium-based cement building materials has spurred considerable research into product characterization and development, including for improving MOC water resistance [26].

Despite these advantageous properties, the application of MOC is not widespread because it reduces strength rapidly on prolonged exposure to water [27]. The durability of MOC could be improved by modifying the composition and introducing various additives [21]. 

**Figure 1:**
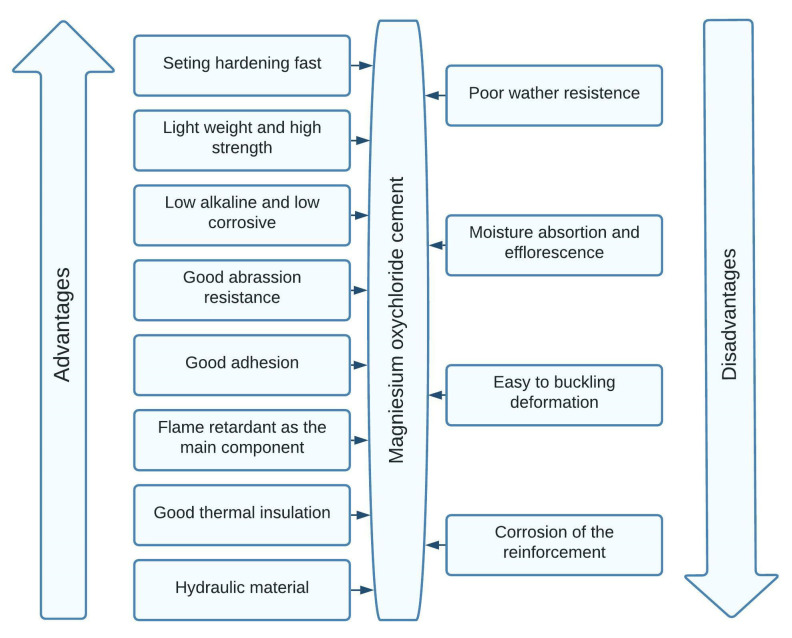
Advantages and Disadvantages of Magnesium Oxychloride Cement.

In order to visualize the main advantages and disadvantages of magnesium oxychloride cement better, Figure 1 was elaborated. It can be seen that MOC cement has many excellent performances such as rapid hardening rate, very high strength, good cohesiveness, shaping convenience, easy to be colored, good fire-resistance and the ability to resist high temperature [28]. 

The rapid setting and hardening make it ideal to be used in the case of constructions jobs that require little time for executions, such as repairing infrastructure, for instance, highways and airport runways [8]. This propriety makes the MOC cement a good solution to be used together with ordinary Portland cement to obtain a rapid hardening concrete mixture, as shown in [29]. The density of Magnesium oxychloride cement is 1600–1800 kg/m^3^, only about 70% of Portland cement. The compressive strength is generally more than 50 MPa after curing 28 days, which can even break through 200 MPa after adding a modifier, in which case, the rupture strength is more than 10 MPa, and the bending stress learning performance is better [30].

The advantage of lower alkalinity makes the MOC cement a good solution to be used with glass fibers [31] without the aging problem, which is very common when glass fibers are mixed with ordinary cement. MOC cement is also good for mixing with wood particles and sawdust to make wood-like composites and building products [8].

The good abrasion resistance [30] combined with good mechanical proprieties [32] makes it possible to use MOC cement for making industrial floors, grinding wheels and functional panels [19]. Another use of MOC cement is in the production of magnesium oxychloride cement bricks for fine polishing of porcelain stoneware tiles [33]. As it is an air-hardening inorganic adhesive, the MOC cement has good bonding strength to different substances [24].

Another important advantage of the MOC cement is referring to its good fire protection proprieties, with a flame retardant as the main component [23]. These proprieties make it suitable to be used in different mixtures to improve the fire protection of the building materials. In the paper [34], it is shown that using MOC cement in the mixture of Laminated Veneer Lumber not only improves the mechanical strength of the material but also suppresses the production of smoke and reduces the production of combustion heat.

The researchers identified some possible utilization of the MOC cement in the production of more healthy building materials. In [35], the authors studied its ability to have good antimicrobial proprieties in combination with silver phosphate. Other researchers address its air purification behavior [19]. 

The multiple advantages of MOC cement make it suitable to be used in combination with other new and promising materials such as graphene. In [36], the researchers studied the influence of the graphene-specific surface area used as a dopant in MOC cement. The researchers [37] analyzed and prepared novel nanocomposites containing carbon-based nanomaterials and MOC cement. The conclusion of this study indicates that “the prepared materials containing graphene, graphite oxide, or a combination of both additives in the total amount of 0.5% by weight of binder material exhibited remarkably enhanced mechanical characteristics such as flexural and compressive strength”. The researchers of the paper [38] published multiple articles analyzing the possibilities of combining MOC cement with graphene, studying the possibility of producing new high strength and water resistance building material [39], or the influence of the mixture preparation technology of the graphene and MOC cement and how this influence its properties [40].

MOC cement also has various other applications, such as the use of composite building materials based on MOC for stabilization or solidification of sewage sludge [41]. Other research directions explore the possibility of obtaining good building materials using seawater [42] or different compounds from the salt lakes [43] and also how the MOC composite responds to the salt attack [44].

Despite its multiple advantages, the use of MOC cement widely is limited because of its important disadvantages. The biggest disadvantage is referring to the poor water resistance. Tests performed by immersing building elements in water revealed that, two months later, the magnesium oxychloride cement specimen had a serious quality loss, as its compressive strength decreased by more than 80% [30]. Other disadvantages appeared from this poor water resistance, such as moisture absorption and efflorescence or corrosion of reinforcement in the reinforced concrete building elements. The poor water resistance of MOC cement has been a barrier to its further commercialization [24]. 

Various solutions were studied and developed to counteract this disadvantage, such as adding various additives and filler into the MOC concrete [45]. For instance, the addition of a small quantity (for example, 1% by weight of magnesia) of phosphoric acid or soluble phosphates can greatly improve the water resistance of MOC [46]. Other researchers found that indeed some additives are very helpful in overcoming the problem of water solubility [47]. In another research, it was shown that using phosphate could react with magnesium to produce insoluble hydrated products such as magnesium phosphate that protected the magnesium cement crystal from decomposing. At the same time, if the quantity of the insoluble phosphates is not high enough, then it does not produce a layer of insoluble phosphates. On the other hand, some studies revealed the transformation from the crystalline to gel-like Phase 5, which was believed to be the main reason for the improved water resistance of MOC when adding phosphate. Although adding additives is an effective way to improve the water resistance of MOC, it increases the cost of producing the material. Therefore, using waste materials should be considered [48].

The curing of the cement was also studied to identify if some solution to diminish the disadvantages can be found. In [49], the curing temperature was analyzed, while in [50], it was shown that “introduction of the high temperature curing at 75 degrees C can lead to significant improvements on compressive strengths of these MOC cement-based composites that had ambient curing at the early age”. The curing process was analyzed together with the effect of and H2O/MgCl2 ratio [51], showing that the “curing temperature on the phase structure and mechanical performance of the MOC cement was closely involved with the H_2_O/MgCl_2_ mole ratio in the MgO–MgCl_2_–H_2_O ternary system”.

The poor water resistance of the MOC cement leads to a series of problems if it is used as a replacement for the ordinary Portland cement in reinforced concrete building elements, especially due to the possibility of reinforcement corrosions. Various studies address these issues; in [52], the researchers addressed that the issue of time depended on the model and time predictions for the reinforcement, while in [53], the researcher analyzed the corrosion and anticorrosion of rebar embedded in the MOC cement concrete. In paper [54], the steel corrosion-induced surface damage evolution of the reinforced MOC concrete through gray-level co-occurrence matrices was analyzed.

At the same time, when speaking about a building materials, we need to consider the existence and the extraction of raw resources. The traditional active magnesia used for MOC materials is calcined from magnesite at 700–900 °C. For example, China is one of the countries with the wealthiest magnesite resources in the world, but even if the resources are rich and the grade is high, the distribution is shallow [43]. This can have a big influence on the production costs and, in the end, on the final price of the MOC cement. 

All of the efforts to counteract the disadvantage of poor water resistance imply the use of various additives in the mixture, and despite the effectiveness of additives in improving the properties of MOC, the cost of the end-materials is unfavorably increased [55]. Thus, in order to develop a more sustainable and cost-effective building material, MOC cement must be mixed with other low-cost materials, in general waste from different sources such as waste-expanded polypropylene-based aggregate [56], waste gypsum [57], agricultural and construction waste [55], granite waste [46] or various wood waste [58] such as waste from the construction and demolition [59], especially waste from concrete timber formwork [7]. 

## 3. Materials and Methods

The research methods used in this study were selected considering the main objective of the research. The collection of the materials and their processing starts with a bibliometric literature search, followed by a scientometric analysis and, in the end, an in-depth discussion. The initial selection of data was performed by interrogating the ISI WoS database with certain keywords, and in the second part, the papers were screened, and all irrelevant papers were removed. 

### 3.1. The Process of Collecting Data

**Figure 2:**
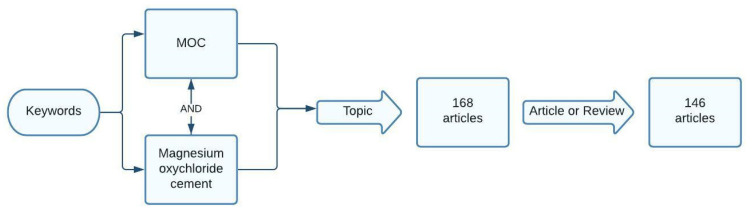
The data collection flow diagram.

The initial data were obtained by interrogating the ISI Web of Science database (ISI WoS). The interrogation of the scientific database was performed using keywords as search criteria (Figure 2). The keywords used were “MOC” and “magnesium oxychloride cement”. In the interrogation process, besides the keywords, the search was oriented on the topic of articles and then filtered to include just paper type “article” or “review”, ignoring the other type of papers such as “conference paper” or “book”. 

The process of collecting the data was performed using the PRISMA method (Preferred Reporting Items for Systematic Reviews and Meta-Analyses). This method was developed by the researchers Moher et al. [60]; they proposed a four-step approach to identify and extract the required data. The first step is the identification of possible data; then, in the screening and eligibility steps, the data were filtered such that all the irrelevant items were removed. At the end of the method, the inclusion step, the final database was formed.

### 3.2. The Extraction and Analysis of the Research Data

The interrogation of the ISI WoS database resulted in a number of papers that were exported in a plain text file format. The method of data extraction was chosen considering the processing process used in this study. For the data procession process, two main software were used. One software is the Bibliometrix software, more precisely the version 3.1. This was developed by Massimo Aria and Corrado Cuccurullo, from the Department of Economics and Statistics, University of Naples Federico II, Italy [61]. The other software was VOSviewer, version 1.6.17. This one was developed by Nees Jan van Eck and Ludo Waltman from Leiden University’s Centre for Science and Technology Studies, The Netherlands.

The data exporting from the ISI WoS database was performed by selecting the full range of resources available. In this sense, in the exported file, each paper has information related to the title of the article, authors, author keywords and the keyword plus, the citation information, the corresponding author, and so on. One of the biggest challenges in performing bibliometric analysis is related to the need to have a certain uniformity of the input data [62]. The interpretation of the results is made according to some network maps or charts generated by the software VOSviewer or Bibliometrix, and thus, the accuracy of the input data is very important. Processing and standardization of the raw data exported from the ISI WoS database were performed manually. This process is very laborious and time-consuming; thus, this is one of the reasons for choosing the ISI WoS database instead of other scientific databases. 

## 4. Results and Interpretation

### 4.1. Number of Publications

**Figure 3:**
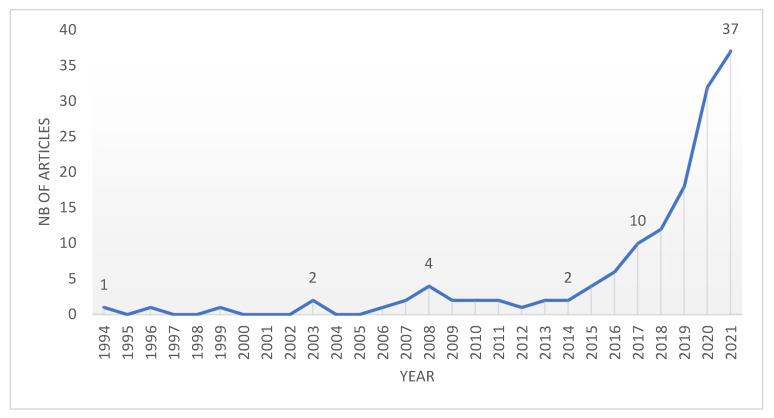
The annual number of publications.

For this part of the research, by using the results offered by the ISI WoS database and the Microsoft Excel software was generated a graph with the annual number of published articles on the studied topic (Figure 3), this can be used as an indicator of the interest given by the researchers to this subject. The graph from Figure 3 was generated for the category of articles that resulted from interrogation the database with the keywords. The annual number of publications is taken directly from the data presented by the ISI WoS database. The type of graph uses two axes, the horizontal axis X and the vertical axis Y. The publication years are represented on the *x*-axis, and the number of articles published in each year is represented on the *y*-axis. 

The data presented in Figure 3 indicate an upward trend in the number of annual articles publications and thus an increasing interest in the topic of MOC cement in the last years. In the interrogation of the ISI WoS database using MOC as search criteria, the first paper indexed in the scientific database was in the year 1994. From then until 2014, the annual evolution of the papers was quite a small one; two or none papers were published, with an exception in the year 2008 when four papers were published. From the year 2014, the trend started to increase; in 2017, a number of 10 papers were published, and in 2021, the number of papers reached 37. If we analyze the absolute values, it can be observed that in the last five years, from 2017 to 2021, three times more articles were published than all the papers cumulated until 2017. Therefore, in the last five years, there has been a substantial increase of the interest in the knowledge, development and use of MOC cement as an alternative to ordinary cement. If we analyze this very large increase in recent years in the context of social awareness of environmental issues, we can expect that this trend will continue to grow.

### 4.2. Journal Analysis 

Given the increased interest in the topic of the MOC cement, the researchers focus their attention on this subject and disseminate their results in various journals. By considering the varieties of journals, a source analysis of the papers was performed. When considering the number of articles published in Figure 3, the first 10 journals with the most articles that address the topic of MOC cement are presented.

**Figure 4:**
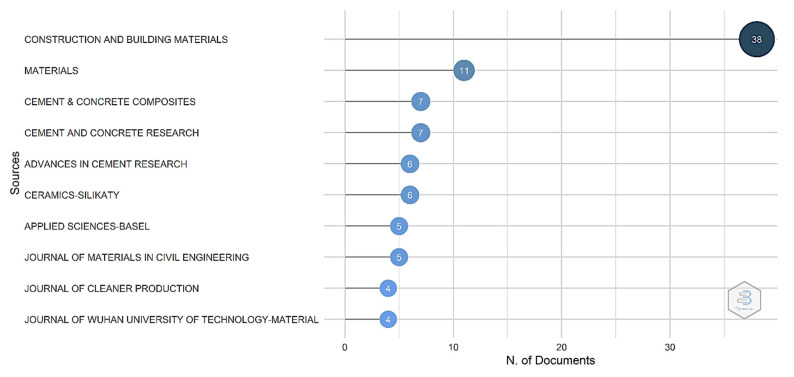
The top 10 journals from the number of publications on the subject of MOC.

The data presented in Figure 4 indicate that the biggest number of articles were published in the journal *Construction and Building Materials*, with 38 papers published. The second journal with half of the number of papers is the journal *Materials,* with 17 papers published. In the third and fourth positions are journals *Cement & Concrete Composite* and *Cement and Concrete Research*, both with seven papers. 

**Figure 5:**
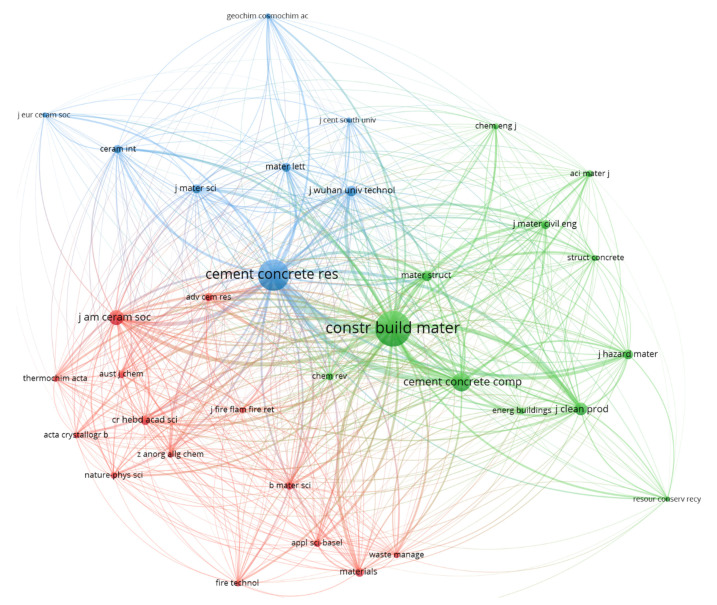
The co-citation network analysis of main sources of articles.

The impact of the journals in the research field was further analyzed with the help of a co-citation analysis map generated with the VOSViewer software (Figure 5). The graphical representation of the map is formed by a series of bubbles or nodes and some connecting lines. Each journal that meets the threshold is represented by a bubble. The map is generated considering the number of co-citations for each journal and the interconnections between them. The size of the bubble is established according to the number of co-citations; thus, the bigger the size, the bigger the number of citations. The interaction between the journals considering the co-citations is represented by a series of lines [63]. For better visualization of the data, the map uses colors. Each journal is grouped in a category with other journals by the similarities of the published articles; therefore, journals sharing the same colors are considered to have similar concerns related to the studied subject. Another aspect observed on the map is the position of journals; with regard to this aspect, the journals cited with a higher frequency are represented close to the center of the map. 

The input data used to generate the map from Figure 5 are based on a total of 1111 sources, then the minimum number of citations for a journal to be included in the map was set to 20. A number of 35 journals with this limitation meet the threshold. By considering the interactions and links between journals, the software generated three distinct clusters. The repatriation of journals as cluster items formed the biggest cluster, the red with 14 items, followed by the green cluster with 12 items and the blue cluster with 8 items.

The co-citation network map highlights two main journals from the MOC cement topic of research. The journal most visible on the map is *Construction and Building Materials,* from the green cluster, with a number of 808 citations and a total link strength of 15,386. The second journal highlighted on the map is *Cement and Concrete Research*, from the blue cluster, with 629 citations and a total link strength of 12,285. Both of the journals are also situated in the top journals by the number of published papers. Other journals highlighted on the map are, from the green cluster, the journal *Concrete & Cement Composite*, with 230 citations and a total link strength of 5924, and from the red cluster, the *Journal of the American Ceramic Society*, with 149 citations and a total link strength of 3135. 

### 4.3. The Countries Scientific Productions

**Figure 6:**
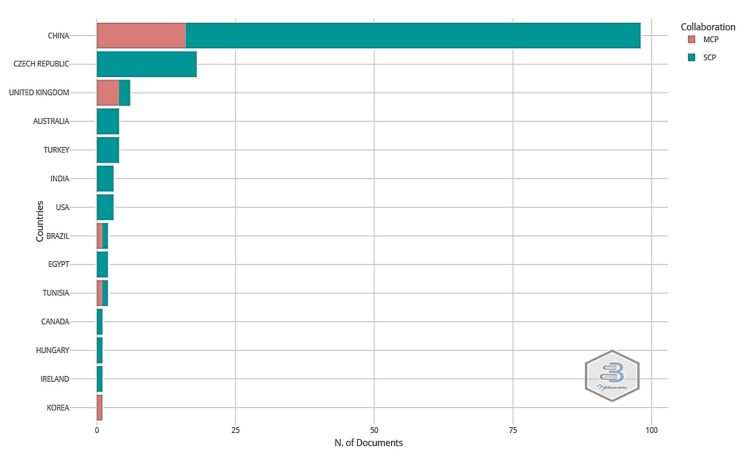
The distribution of papers according to the corresponding author’s country. MCP—multiple country production; SCP—single country production.

The interest in the subject of MOC cement has increased substantially in the last five years. For a better understanding of whether this is just a local subject or is something approached globally, Figure 6 presents the distribution of papers according to the correspondence author’s country. The graph was generated using Bibliometrix software. The graph shows the number of documents on the abscissa and the corresponding author’s country in the ordinate. Represented articles that have been elaborated through international collaboration, with authors from different countries, are noted with MCP, and articles with all authors from the same country are presented with SCP. 

The distribution of the papers according to the corresponding author and country reveals that the study of MOC cement is performed only in a few countries in the world. From the data presented in Figure 6, it can be observed that the most productive country in this field is China; its researchers published 98 papers out of the 146 articles identified after the interrogation of the ISI WoS database. From these 98 papers, only 16 of them were developed by international research teams; the rest of the papers (82) were written by Chinese researchers. The next country with an implication in the MOC cement research field is the Czech Republic, with 18 published papers. In this case, all of the papers were written by Czech researchers. The analysis of the countries’ scientific productions revealed that more than 65% of the total research field is dominated by Chinese researchers. 

### 4.4. The Topical Focus in the MOC Cement Field of Research

**Figure 7:**
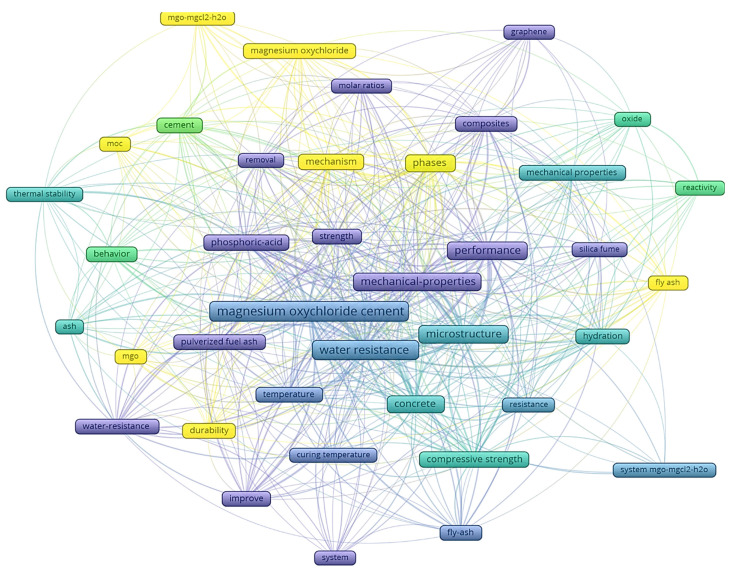
The keywords co-occurrence network map.

The MOC cement field of research offers multiple directions of research, and in order to identify the main ones, a keyword co-occurrence map was generated (Figure 7). The network map was generated using the VOSviewer software. In the case of a journal co-citation map, the visual representation of the keyword co-occurrence is a network map. The principles of generating the map are the same as in the journal co-citation case; the map highlights the most frequently used keywords and their interconnections. In ref. [63], the co-occurrence concept is described as an indicator for a certain similarity and a close relation between the used keywords when the words co-occur in documents. As shown in the [64,65], the output of the keywords co-occurrence analysis not only identifies the most used keywords by the authors but also can indicate a certain trend or pattern in the research area. 

The total number of keywords identified in the selected papers is 596, and they are used as the starting base for the map. The threshold selected for a keyword to be included in the map was set at a minimum of five occurrences, and thus a number of 41 keywords meet the threshold. 

The network map highlights the keyword “magnesium oxychloride cement” in the center of the map with a total occurrence of 70, a total link strength of 281 and average citations of 13.03. From the same cluster, another keyword highlighted is “water resistance” with an occurrence of 59, a total link strength of 296 and average citations of 13.68. Other keywords such as “mechanical-proprieties” can be observed in the center of the map, with an occurrence of 37, a total link strength of 201 and average citations of 7.49. With an occurrence of 36, the keyword “performance” is situated near the “mechanical-proprieties”, with average citations of 9.39 and a total link strength of 194.

Other keywords identified in the co-occurrence network map are “strength”, “phases”, microstructure”, “compressive strength”, “behavior” or “curing temperature”. The keywords analysis revealed that most of the keywords used in the field of MOC cement indicate that the major preoccupations are oriented to better understand the proprieties of the material, the main characteristics and possibilities of using it in diverse construction systems.

#### Trend Topic Analysis

**Figure 8:**
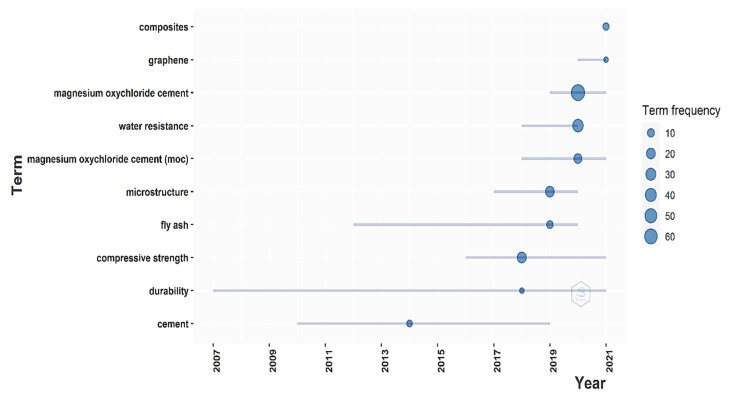
The trend topics plot.

The author’s keywords were further on analyzed, and a trend topic plot (Figure 8) was generated using the Bibliometrix software. The generation of the plot was performed considering a minimum word frequency of five and three words considered per year. The trend topic plot consists of a representation of the main terms used on the vertical axis and the years on the horizontal axis. The term frequency is represented by a bubble. The size of the bubble indicates the time frequency; thus, the bigger the size of the bubble, the bigger the frequency of using that term. A series of lines that are used to highlight the years when that keyword was used can also be observed in the plot. 

The trend topic plot reveals the evolution of the annual approaches regarding MOC cement. By considering the small number of papers published before 2018, the software only found a most frequently used term in 2014, which is cement. Later, in 2018, terms such as “durability” or “compressive strength” started to be used. “Microstructure” and “fly ash” were the most used terms in 2019, while “magnesium oxychloride cement” only appeared as the most frequently used keyword in 2020. At the same time, the plot presents “water resistance” as an important subject approached in the last year. If we consider that this is the biggest limitation of using MOC cement, then the water issues are natural to be approached. In the last year, 2021, the other two terms, “composites” and “graphene”, appear to be more studied. This does not come as a surprise as the MOC cement is studied to be used in different composite building products and graphene is maybe one of the future breakthrough materials that can change the way we build.

## 5. Discussions 

In the effort of reducing the CO_2_ emissions generated by the construction industry, solutions that can decrease the use of concrete and better use of wood products are welcome. Wood is a sustainable building material with many advantages and multiple-use possibilities. Wood is an inhomogeneous material (anisotropic) made up of a large number of plant cells organized into specialized tissues, also called anatomical elements, which are very diverse [66]. They differ in their lifetime and tree functions, shape and size, position in the tree and quantity or number. Many of the cells die during the life of the tree, retaining only the role of ensuring the mechanical strength of the tissues in its composition. The way in which the anatomical elements observable with the naked eye are grouped is called the macroscopic structure [5].

A big challenge of the building industry is to better manage the waste resulting from the production of building materials in various building stages. In [67], it was shown that “proper utilization of sawdust in concrete will conserve the environment by reducing the use of natural resources, reducing the volume of waste material, and reducing CO_2_ emissions”. Waste can be incorporated into the concrete mixture in the form of flying ash. The studies presented in [68] reveal that “the incorporation of fly ash can enhance the workability or fluidity, retard the setting time, and improve the water resistance of the MOC mortars”. Other subject addresses refer to its rheological properties [69], the effect of increasing the resistance of the MOC cement composites under the water attack [70], the influence on the compressive strength [46], the improvement of the water resistance [71] or the anticorrosion effect for the reinforcement [72]. 

Several other researchers identified the advantages and possibility of combining wood and concrete. In the paper of [73], the researchers presented a new material, Wood-Crete, indicating the possibility of using wood waste and concrete products for in-fills for wall panels and hollow blocks or for thermal insulating material. The good properties of the wood wool–cement composite material are highlighted by [74], which shows that “the material presents excellent mechanical, chemical and biological properties. However, the understanding of its mechanical behavior is rather limited”. The incompatibility between wood and cement is analyzed by [6], and it is proven that “the alkaline hydrolysis was found as the most effective treatment for the suppression of inhibitory substances and the highest decrease in mechanical properties of resulting composites”. There is obviously a need to find a proper balance in the wood-and-cement composition, which is quite difficult to determine.

Wood–cement composites are now being investigated and made industrially in many countries in the world, mostly in the form of panels because of their excellent exterior properties [75]. The main difficulty for wood–cement composites manufacturing is the chemical incompatibility between wood and cement; in most cases it is the ordinary Portland cement that inhibits cement setting and hardening [7]. The inhibitory substances mainly include some sugars, part of hemicelluloses and their degradation products. The inhibitory degree is affected by many factors, including wood species, location, part of the tree, season during wood-cutting, wood/cement ratio, type of cement, storage condition and other factors [76].

Studies have shown that wood-MOC composites prepared with a higher wood fiber content had a lower thermal conductivity, higher bending resistance, higher residual bending resistance after exposure to high temperatures and water immersion and a better noise reduction effect. Even if the water absorption increased with an increase in wood fiber content, it could still be considered low [48]. The proprieties of the MOC cement make it suitable to be used in the composition of building elements with good sound absorption levels [76]. Other identified solutions propose the use of MOC cement and wood to produce lightweight composite building materials [8] through the process of extrusion. 

At the same time, MOC cement can be used with great results as an alternative to the adhesive solutions used in wood building materials. Several studies are addressing this issue and proposed different solutions, as in [75], where it is proposed to use MOC cement inorganic adhesives are presented as an effective and sustainable binder for plywood applications. The study [24] focused on the preparation of an eco-friendly and high-performance MOC-based formaldehyde-free adhesive based on an organic–inorganic hybrid structure, showing that MOC cement deposited on the fibers’ surface was connected by the hydrogen bonds in wood, which improved the mechanical properties of wood-based composites. In addition, the use of MOC improved the flame retardancy of the plywood. 

By using MOC cement in the composition of wood panels, studies showed a decrease in greenhouse gas emissions. In [7], it was shown that “greenhouse gas emissions associated with wood-MOC board incorporating incinerated sewage sludge ash was 71% lower compared to plywood production, and comparable to the resin-based particleboard. Moreover, the human toxicity of the wood-MOC board was 58% lower compared to the conventional resin-based particleboard production due to the latter uses a large amount of highly toxic organic resin”.

Other approaches in using MOC cement combined with wood can be identified in [8], where it is shown that “the specific dry densities of the wood–MOC cement composites were close to 1.0 and they were nailable like hard natural wood. Their flexural strength decreased as temperature increased. By replacing 50% sawdust in weight by perlite, the composite exhibited less die swell and better performance in resisting high temperature”.

The research results indicate that the field of magnesium oxychloride cement has been presenting more and more interest in the last years. The annual evolution of the number of published articles indicates a big increase in the last five years. An interesting aspect is a fact that in the last five years, from 2017 to 2021, 109 articles were published, three times more than the total number of 33 articles published before 2017. Considering that in the last years the awareness of the impact of human activities on the environment is more and more felt and that world leaders enroll in making efforts to reduce the negative impact and reduce the global warming phenomenon, sets the premises of a future increase in the interest in more eco-friendly solutions to build. 

The increased interest in MOC cement as building materials led to the dissemination of the results in various journals. From this point of view, the journals with the biggest number of publications are *Constructions and Building Materials* and *Materials*. In the co-citations analysis, besides the journal *Construction and Building Materials*, which is the biggest on the map, journals such as *Concrete and Cement Research* and *Concrete & Cement Composites* are other journals highlighted as being more influential in the research field. An interesting aspect revealed in this study is that more than 65% of the total scientific production is produced by researchers from China. In the second place from the number of papers, but far away, are the researchers from the Czech Republic. 

From the main subjects addressed in the field of MOC cement, the trend topic analysis revealed that magnesium oxychloride cement is only a keyword that was most frequently used in the year 2020 and that the first keyword mostly used was cement in 2014. In the last year, other words are becoming more and more used alongside the MOC cement, such as “composite” or “graphene”. Both of the two directions present a big interest and a big potential to generate new building materials that will decrease the negative impact of the construction industry on the environment. 

## 6. Conclusions

The purpose of this paper was to investigate if MOC cement can be a good solution to produce wood–cement building materials that can help decrease the negative impact of the construction industry on the environment. The main conclusions resulting from this study can be formulated as:

o In the last five years, from 2017 up to 2021, the interest for MOC cement increased substantially; in this time, more than three times more papers were published than all of the papers cumulated before 2017;
o The MOC cement presents a lot of advantages, in many ways it exhibits properties superior to standard Portland cement. The biggest disadvantage that acts as the major limitation for its widespread use is its poor water resistance;
o The MOC cement presents very good compatibility with wood, and by using wood waste in the mixture, the positive effect on the environment can be increased;
o A big advantage of using MOC cement in combination with wood is given due to the natural color of the MOC cement, which is yellowish, so it is closer to the color of many natural wood species;
o The main journals publishing papers about this subject are *Construction and Building Materials*, the journal *Materials*, the journal *Cement&Concrete Composite* and the journal *Cement and Concrete Research*;
o An interesting aspect related to MOC cement is that more than 65% of the studies in the field are conducted by Chinese researchers. They are followed, at a big distance, by researchers from the Czech Republic, where published papers are written only by Czech researchers;
o The MOC cement is only the most frequently used keyword from 2020, indicating that it is a new material with a big potential to be further discovered and used to obtain various composite building materials.

Some future research directions can be explored further when discussing wood and MOC composite building materials. Most of them are related to an effort of realizing more experimental studies to identify the proper ratio mixt between wood and MOC cement. Some price analysis must also be performed in order to develop a building material with more widespread use. 

The general conclusion of this research can be considered that there is a big potential of using magnesium oxychloride cement as a replacement for Portland cement, especially in the mixture of composite building materials where ordinary cement has some incompatibilities. Decreasing the negative impact on the environment of the building sector can start by using more sustainable materials such as wood and also using wood waste from different sources. Using wood and MOC cement is a good alternative to produce sustainable and more eco-friendly building materials with the same or better proprieties than concrete.

## Data Availability

Not applicable.
